# A randomized controlled trial of a supervised self-administered program for chronic plantar fasciitis

**DOI:** 10.1186/s12998-025-00624-w

**Published:** 2025-12-23

**Authors:** Vitsarut Buttagat, Yadanuch Boonyaratana, Sujittra Kluayhomthong, Sulukkana Noiprasert, Petcharat Keawduangdee, Pattanasin Areeudomwong

**Affiliations:** 1 Department of Physical Therapy, School of Integrative Medicine, Mae Fah Luang University, Chiang Rai, 57100 Thailand; 2 Department of Traditional Chinese Medicine, School of Integrative Medicine, Mae Fah Luang University, Chiang Rai, 57100 Thailand; 3 Research Group On Smart Integrative Medicine and Technology Sustainability, Mae Fah Luang University, Chiang Rai, 57100 Thailand

**Keywords:** Plantar fasciitis, Strengthening exercises, Stretching exercises, Integrated self-administered program

## Abstract

**Background:**

Plantar fasciitis (PF) is the most frequent cause of chronic heel pain. Conservative interventions, including strengthening and stretching exercises as well as massage, are commonly recommended as first-line management. However, the effectiveness of an integrated self-administered program (IA) for patients with chronic PF remains unclear. Therefore, this study aimed to evaluate the effectiveness of a supervised IA compared with a self-care leaflet in individuals with chronic PF.

**Methods:**

Sixty-four participants with chronic PF (47 females, 17 males)were randomly allocated to either the intervention group (n = 32; mean age 60.3 (SD 3.7) years) or the control group (n = 32; mean age 56.9 (SD 5.4) years). The intervention group engaged in a multi-component program that included foot and ankle strengthening, active stretching, and self-administered Thai massage using a self-treatment stick. The program was performed three days per week for four weeks in a community-based setting under therapist monitoring to ensure safety and adherence. In contrast, the control group received an educational leaflet on self-care. Pain intensity (measured using the visual analogue scale), pressure pain threshold, ankle dorsiflexion range of motion, and foot and ankle ability measure (FAAM) were measured at baseline, at the end of the fourth week (week 4), and one month after the intervention period ended (week 8). The primary outcome for this study was designated as pain intensity.

**Results:**

After adjusting for baseline values, the intervention group demonstrated significantly greater improvements in pain intensity (Mean Difference: − 2.5 [95% CI − 3.5, − 1.5]), pressure pain threshold (5.7 [4.8, 6.6]), ankle dorsiflexion range of motion (5.2 [3.2, 7.3]), and FAAM (15.3 [9.6, 20.9]) compared with the control group (all *p* < 0.05). These effects were maintained at week 8, indicating sustained benefits of the integrated supervised self-administered program.

**Conclusions:**

This integrated supervised self-administered intervention may serve as a practical and effective approach to self-care in the management of chronic PF.

**Trial registration:**

This study was prospectively registered at Thai Clinical Trials Registry (ID: TCTR20240923001).

**Supplementary Information:**

The online version contains supplementary material available at 10.1186/s12998-025-00624-w.

## Background

Plantar fasciitis (PF) is the most common cause of chronic heel pain, typically affecting individuals aged 40–70 years, with a higher prevalence in women [1, 2]. Although traditionally viewed as an inflammatory disorder, current evidence suggests that it may be more accurately described as plantar fasciosis due to chronic degenerative changes rather than a primarily inflammatory process [3]. Clinically, PF presents as dull, aching pain at the inferior heel, often most noticeable during the first steps in the morning or after inactivity, with symptoms frequently returning during prolonged weight-bearing [4, 5]. The underlying pathogenesis is believed to involve degenerative changes and repetitive mechanical loading at the calcaneal attachment of the plantar fascia, contributing to collagen disruption and structural breakdown [6]. Limited ankle dorsiflexion related to gastrocnemius-soleus tightness may further increase plantar fascia tension by altering calcaneal alignment [7]. Weakness of intrinsic foot muscles and lower-leg muscles involved in foot and ankle function has also been associated with increased plantar pressure, suggesting a potential role for muscular deficits in PF development [8, 9].

Diagnosis is typically established through clinical history and physical examination, with imaging used only in atypical cases [6, 10]. Treatment options range from conservative therapies to surgical procedures [11, 12]. Conservative interventions such as stretching, strengthening, massage, paraffin therapy and therapeutic physical agents are widely recommended as first-line management and report success rates exceeding 70 percent [5, 11–14].

Bolgla and Malone indicated that effective management of PF should focus on restoring foot and ankle strength and flexibility to improve plantar fascia extensibility, joint mobility and overall biomechanics [15]. Consistent with this, Koc et al. highlighted the role of manual therapy and therapeutic exercise, including massage, targeted stretching and strengthening, in improving flexibility, mobility, pain and overall function [16]. Cleland et al. further demonstrated that combining manual therapy with therapeutic exercise resulted in greater symptom improvement than exercise with electrophysical modalities alone [17].

Stretching exercises are frequently prescribed to enhance flexibility and joint mobility. Stretching of the gastrocnemius-soleus complex is particularly effective in PF, as it reduces muscle tightness, decreases plantar fascia tension and helps restore normal ankle dorsiflexion range of motion [16, 18]. This technique is among the most widely recommended components in PF rehabilitation programs [19].

Strengthening exercises aim to improve muscle strength and endurance, and PF rehabilitation should target both extrinsic and intrinsic foot and ankle musculature [20, 21]. Strengthening the toe flexors and plantar flexors has been shown to reduce pain and improve functional performance, including gait [20–22].

Thai massage is widely practiced in Thailand and is used to promote physical health and psychological well-being [23–28]. Evidence indicates that Thai massage can improve circulation, increase tissue flexibility, reduce pain and induce relaxation across different patient groups [26–29]. In individuals with PF, Poonsuk et al. reported that Thai massage was more effective than elastic taping and stretching in reducing heel pain and improving functional performance [30].

Nevertheless, although these evidence-based interventions are commonly provided in hospital or clinical settings, they may be costly or not readily accessible to all patients. Therefore, clinical practice should emphasize affordable, evidence-based strategies that can be safely performed by patients with basic professional guidance, such as self-massage, stretching, and strengthening exercises, to support long-term independent symptom management in individuals with PF. Building on this need, the present study investigated the effectiveness of an integrated supervised self-administered (IA) program comprising foot and ankle strengthening, active stretching, and self-administered Thai massage, compared with a self-care leaflet, on pain intensity, pressure pain threshold, ankle dorsiflexion range of motion, and the Foot and Ankle Ability Measure (FAAM) in patients with chronic PF.

## Methods

### Study design

This study was a randomized, controlled trial with intention-to-treat analysis performed between November 2024 and June 2025 in Chiang Rai province, Thailand. The study design was approved by the Mae Fah Luang University Ethics Committee on Human Research (EC 24113-25), and the study was prospectively registered at the Thai Clinical Trials Registry (TCTR20240923001).

### Setting

The present study was conducted at the community halls of the Nang Lae sub-districts in Chiang Rai province, Thailand.

### Participants

Individuals with chronic PF were recruited from Chiang Rai province, Thailand through poster advertisements. Eligible participants were male or female adults aged 40–70 years who presented with the following: (1) sharp heel pain that worsened with weight bearing, particularly during the first steps after waking in the morning; (2) a current pain intensity of 3 or higher on a 0–10 cm visual analog scale; (3) difficulty with heel movement, defined as pain or stiffness during heel raising or lowering movements; (4) the ability to understand and comply with the study procedures and (5) symptom duration of at least 3 months to meet the definition of chronic PF. The exclusion criteria were (1) a history of neurological disorders, such as cerebrovascular accidents, spinal cord injuries, or other conditions that impair the ability to perform daily activities; (2) a recent lower-extremity fracture or a previous fracture that resulted in residual deformity, altered weight-bearing mechanics, or persistent functional impairment; and (3) inflammatory conditions characterized by pain, swelling, redness, or heat in the lower extremities.

The required sample size was determined using data from our pilot study, in which pain intensity measured by the 0–10 cm visual analogue scale served as the primary outcome. The pilot compared an IA program (n = 10) with a control condition (n = 10), yielding a mean between-group difference of 1.3 points, with standard deviations of 1.14 in the IA group and 1.72 in the control group. Using these estimates, a two-sided significance level of 0.05 and a statistical power of 90% indicated that 27 participants per group would be required to detect a difference of this magnitude. To accommodate an anticipated dropout rate of 15%, the final target sample size was set at 32 participants per group.

### Randomization

After baseline assessments were completed, participants were randomly assigned to either the IA group or the control group at an allocation ratio of 1:1. Randomization was performed using a block allocation method with block sizes of two, four, and six to ensure equal probability of assignment and balanced group sizes. A research assistant who was not involved in participant recruitment, intervention delivery, or outcome assessment generated the allocation sequence and prepared the sealed, opaque envelopes. The same research assistant also opened the assigned envelope after baseline assessment and informed the participant of their group allocation.

### Interventions

Both groups received an educational leaflet on basic self-care, which included general information about PF, its symptoms, risk factors to avoid, and basic self-care advice limited to warm foot soaking and weight management. The leaflet did not contain any instructions on massage, stretching, or strengthening exercises. The intervention group additionally received an IA program, performed three times per week, 40 min per session, for a total of four weeks. The control group received only the self-care leaflet without the IA intervention.

### An integrated supervised self-administered program group (the IA group)

Participants assigned to the IA group received a multi-component program comprising self-administered Thai massage, stretching exercises, and strengthening exercises. A specially designed device, the self-treatment stick, developed by the principal investigator (Appendix Fig. A1), was used to facilitate both massage and exercise.

**Fig. 1:**
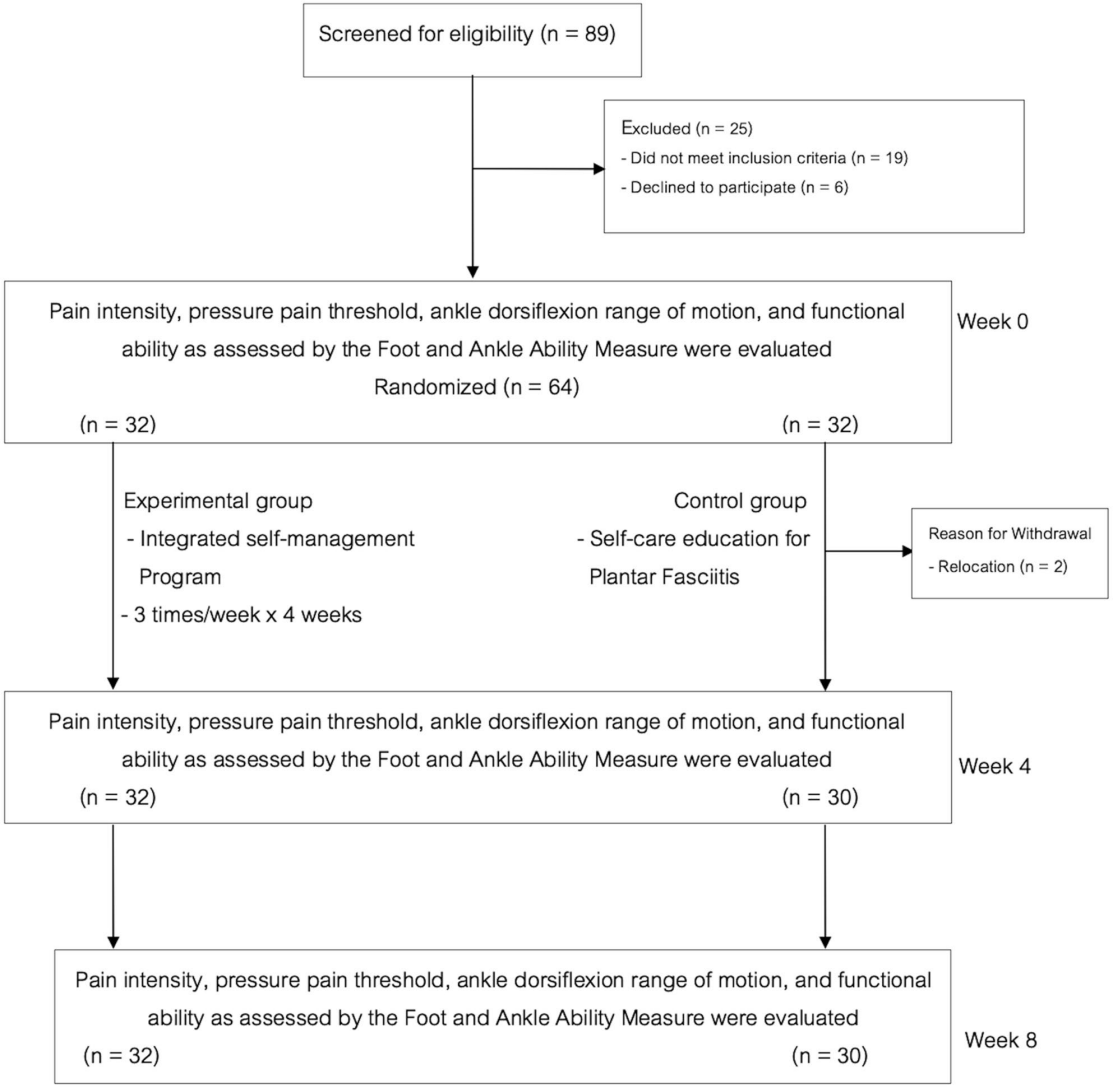
The CONSORT flow diagram

Before entering the program, all participants underwent a structured training session conducted by a licensed physical therapist with more than 18 years of clinical experience. During this session, participants were taught and practiced every component of the study protocol, including self-administered Thai massage, stretching, and strengthening exercises, until they could perform all procedures independently, accurately, and safely. The training took place at the community halls of the Nang Lae sub-district in Chiang Rai, Thailand.

The intervention was conceptualized as a supervised self-administered program. This approach emphasized participant autonomy and skill acquisition while maintaining ethical and safety oversight. Although all treatment sessions were conducted at the same community setting and monitored by a physical therapist, the active components of treatment, including self-administered Thai massage, stretching, and strengthening, were performed entirely by the participants themselves. The therapist’s role was limited to observation and ensuring adherence to the study protocol, preventing potential harm, in accordance with ethical requirements for human research. This framework demonstrates the feasibility of applying a structured self- administered strategy for PF within community-based contexts. The intervention included three main components, as follows.

*Self-administered Thai massage* The participants performed self-massage on the plantar surface of the foot for ten minutes using the self-treatment stick (Appendix Fig. A2). Pressure was applied along specific lines and points (Appendix Fig. A3 in accordance with traditional Thai massage techniques described by Eungpinichpong [31]. The intensity of pressure was increased until a mild sensation of discomfort, corresponding to the pain pressure threshold, was perceived. At each point, pressure was maintained for five seconds before being released, and this procedure was repeated approximately five times along each line.

*Stretching exercises* The participants performed gastrocnemius and soleus stretches using the self-treatment stick while seated. Each stretch was held for 30 s and repeated three times. In the gastrocnemius stretch (Appendix Fig. A4), the participants were seated on a chair with the knee fully extended. The self-treatment stick was used to assist stretching of the gastrocnemius muscle. While holding both ends of the stick, the participant placed the forefoot on the side of the massage knob and pulled the handles toward the body until a strong but pain-free stretch was felt in the calf. The stretch was maintained for 30 s and repeated three times. In the soleus stretch (Appendix Fig. A5), the procedure was identical to the gastrocnemius stretch, except that the knee was slightly flexed while seated. Each stretch was held for 30 s and repeated three times.

*Strengthening exercises* Two isometric strengthening protocols were performed. In the plantar flexor strengthening (Appendix Fig. A6), the participant was seated on a chair with the self-treatment stick positioned under the forefoot. While holding both ends of the stick, the participant was instructed to press the forefoot downward against resistance provided by the device. Each contraction was held for 15 s and repeated 15 times per set, with a total of three sets. All contractions were performed at the maximum effort tolerated without pain. In the toe flexor strengthening (Appendix Fig. A7), in the same seated position, participants used all five toes to grip the self-treatment stick. They were instructed to squeeze the device with maximal effort while ensuring the contraction remained pain-free. Each contraction was held for 15 s and repeated 15 times per set for a total of three sets.

### Control group

The participants in the control group received only an educational leaflet on basic self-care for PF management. After completing the study, all control participants were offered the same IA program as the intervention group.

### Outcome measures

All outcome assessments were conducted at baseline, the end of the treatment program (week 4), and one month after the end of the treatment program (one-month follow-up: week 8) by the second research assistant who was independent of the trial. Blinding was applied only to the assessor who measured the secondary outcomes (pressure pain threshold and ankle dorsiflexion range of motion). Because the primary outcome (pain intensity) and FAAM were self-reported by participants, blinding of these measures was not feasible.

### Primary outcome measures

#### Pain intensity

The pain intensity was measured using a 10-cm visual analogue scale and ranged from 0 (“no pain”) to 10 (“worst pain imaginable”). Participants were instructed to mark the point that best represented their current level of pain. The visual analogue scale has been shown to be highly reliable (r = 0.99) [32] and valid [33].

### Secondary outcome measures

#### Pressure pain threshold

The pressure pain threshold was assessed following the procedures described by Fisher [34]. After receiving a detailed explanation of the protocol, the participants lay in a prone position. The assessor identified the most tender point on the plantar surface and marked the site with a washable pen. Then, the marked location was traced onto a transparent sheet to ensure accurate repositioning in subsequent assessments. A pressure algometer was then applied perpendicularly to the marked point at a constant rate of approximately 1 kg/s. The participants were instructed to signal the moment they first perceived pain, which was recorded as the pressure pain threshold. Measurements were taken twice, with a three-minute interval, and the mean value was used for analysis. This method has been shown to demonstrate high reliability [35].

#### Ankle dorsiflexion range of motion

Ankle dorsiflexion range of motion was assessed using a standard goniometer. The participants were seated on a table with the tested knee fully extended and the contralateral leg hanging off the side. The measurement procedure followed the method described by Tavares et al. [36]. This technique has demonstrated excellent reliability (r > 0.91) [37].

#### Foot and ankle ability measure (FAAM)

Functional ability of the foot and ankle was assessed using the Thai version of the FAAM [38]. The FAAM consists of 21 items for activities of daily living and 7 items for sports activities, each scored from 0 (unable to perform) to 4 (no difficulty). In the present study, only the activities of daily living subscale was used. Lower scores indicated greater functional limitation. The Thai version has demonstrated good reliability and validity [38].

### Adverse events

Potential adverse events associated with the intervention were closely monitored during the four-week intervention period. The participants were asked on a weekly basis about any abnormal symptoms or discomfort following treatment, and all reported events were documented for research purposes.

### Statistical analysis

Statistical analyses were conducted using SPSS software (version 20.0; IBM, Armonk, NY, USA). Normality was assessed with the Shapiro–Wilk test. Continuous data were presented as mean ± standard deviation (SD), while categorical data were presented as frequency counts (%). Intra-group comparisons were analyzed using repeated-measures analysis of variance (ANOVA), with Bonferroni-adjusted post-hoc tests applied when significant differences were detected. Inter-group comparisons were performed using analysis of covariance (ANCOVA) while adjusting for baseline scores as covariates. Significance was set at α = 0.05.

## Results

A total of 89 patients were initially recruited. Twenty-five did not meet the inclusion criteria, and the remaining 64 patients with PF were randomly assigned to either the IA group or the control group. During the intervention, two patients in the control group withdrew because they moved to another city. Consequently, 62 patients (32 in the IA group and 30 in the control group) completed the study. Adherence to the intervention protocol was high. All participants in the IA group attended all 12 supervised sessions as scheduled, and no deviations from the prescribed program were reported. Figure 1 presents the CONSORT flow diagram, while Table 1 summarizes the baseline participant profiles.

**Table 1:** Baseline participant profiles

Characteristic	Randomized (n = 64)	
	IA (n = 32)	Control (n = 32)
Age (years), Mean(SD)	60.3 (3.7)	56.9 (5.4)
Sex; number of females (%)	24 (75)	23 (71.88)
Weight (kg), Mean(SD)	56.2 (7.5)	57.8 (8.5)
Height (m), Mean(SD)	1.56 (0.14)	1.58 (0.08)
Body Mass Index (kg/m^2^), Mean(SD)	23.6 (6.2)	23.3 (3.8)
Duration of the heel pain episodes (month), Mean (SD)	13.2 (4.7)	14.1 (4.9)

*IA* Integrated self-administered program, *SD* Standard deviation

### Effect of the intervention

As summarized in Table 2, after adjusting for baseline values, the between-group analysis revealed that the IA group achieved significantly greater improvements in pain intensity, pressure pain threshold, ankle dorsiflexion range of motion, and FAAM compared with the control group. These differences were evident at both week 4 and week 8 (*p* < 0.05).

**Table 2:** Changes over time (repeated measures ANOVA) and adjusted treatment effects on all outcome measures (ANCOVA)

Outcome	Groups	Within-group comparisons			Between-group comparisons	
		Baseline mean (SD)	Week 4 mean (SD) (Unadjusted)	Week 8 mean (SD) (Unadjusted)	Adjusted mean difference [95% CI]^A^ (Week 4)	Adjusted mean difference [95% CI]^A^ (Week 8)
Pain intensity (0–10 cm VAS)	IA	6.8 (1.7)	3.3 (1.9)*	2.7 (2.3)*	− 2.5^#^ [− 3.5–− 1.5]	− 2.9^#^ [− 3.9–− 1.9]
	Control	6.3 (1.8)	5.6 (2.2)	5.4 (2.1)*		
Pressure pain threshold (N/cm^2^)	IA	4.0 (2.7)	9.0 ± 2.8*	9.5 (3.0)*	5.7^#^ [4.8–6.6]	6.1^#^ [4.0–7.2]
	Control	4.9 (2.9)	3.9 ± 2.5*	3.9 (2.3)*		
Ankle dorsiflexion range of motion (degrees)	IA	6.4 (4.6)	13.0 (4.8)*	13.2 (4.1)*	5.2^#^ [3.2–7.3]	6.4^#^ [4.7–8.2]
	Control	8.5 (5.2)	8.7 (4.3)	7.6 (3.6)		
The Foot and Ankle Ability Measure (FAAM score, 0–100)	IA	52.2 (15.7)	69.7 (11.0)*	73.4 (10.6)*	15.3^#^ [9.6–20.9]	19.7^#^ [14.3–25.2]
	Control	51.5 (10.4)	54.2 (13.4)	53.5 (12.6)		

*IA* Integrated self-administered program, *VAS* Visual analogue scale, *SD* Standard deviation

*Significant difference from baseline within the group (*p* < 0.05), derived from Repeated Measures ANOVA

^A^Adjusted mean difference and 95% confidence intervals were derived from the Analysis of Covariance (ANCOVA) models, adjusting for baseline values

^#^Significant difference between groups (*p* < 0.05), derived from ANCOVA, CI = Confidence interval

Within-group comparisons further showed that, relative to baseline, the IA group demonstrated significant reductions in pain intensity and significant increases in pressure pain threshold, ankle dorsiflexion range of motion, and FAAM at both week 4 and week 8 (*p* < 0.05). By contrast, the control group showed improvement only in pain intensity at week 8, whereas pressure pain threshold values decreased at both week 4 and week 8 (*p* < 0.05) (Table 2).

### Adverse events

In the present study, no adverse events were observed in either the IA or control groups, with the exception of one participant in the IA group who reported mild soreness in the gastro-soleus muscle after the first treatment session. The symptom resolved spontaneously within 24 h. The participant declined further treatment for this complaint, as the discomfort was minimal and improved rapidly. Importantly, no recurrence of soreness was reported by this participant for the remainder of the intervention program.

## Discussion

To our knowledge, this was the first clinical trial to evaluate the effectiveness of an IA combining self-administered Thai massage, stretching, and strengthening exercises using a self-treatment stick on pain intensity, pressure pain threshold, ankle dorsiflexion range of motion, and FAAM in patients with chronic PF. When compared with the control group, participants in the IA group demonstrated significantly greater improvements in pain intensity, pressure pain threshold, ankle dorsiflexion range of motion, and FAAM after four weeks of intervention, and these benefits were maintained at the eight-week follow-up. The sustained superiority of the IA group over the control condition underscores the longer-term efficacy of this approach beyond basic self-care education. Additionally, the magnitude of change observed across all outcome measures suggests that the improvements were not only statistically significant but also of a meaningful size, exceeding thresholds commonly reported in related musculoskeletal research [39–42]. Comparisons of effect sizes across studies should be interpreted with caution, given the methodological differences among trials; however, the magnitude of change reported here aligns with those documented in similar multimodal interventions for PF [17, 43, 44].

These findings are consistent with the recommendations of Fraser et al. [45], who advocated the use of manual therapy in combination with stretching and strengthening exercises for patients with PF. Likewise, the present results support the conclusions of Koc et al. [16], who emphasized that manual therapy may be effectively complemented by stretching and strengthening exercises in PF management. Importantly, the present study adds to this body of evidence by demonstrating that a structured, supervised, self-administered program yielded superior outcomes compared with self-care alone, suggesting that such an approach can support symptom management in community settings while maintaining adherence and safety through minimal professional monitoring. This supports the feasibility and potential clinical value of incorporating supervised self-administered strategies into conservative PF care.

These findings are also consistent with studies highlighting the benefits of multimodal conservative interventions targeting soft-tissue mobility and neuromuscular function. For instance, Cleland et al. [17] reported that combining manual therapy with therapeutic exercise was superior to exercise plus electrophysical modalities. Saban et al. [43] similarly found that integrating deep massage, neural mobilization, and self-stretching was more effective for plantar heel pain than conventional care. Furthermore, Renan-Ordine et al. [44] demonstrated that adding manual trigger-point therapy to a self-stretching protocol yielded superior short-term results. Collectively, these studies reinforce the potential benefits of combined therapeutic approaches.

The improvements observed in this study may be attributable to several complementary mechanisms arising from the combination of self-administered Thai massage, stretching, and strengthening exercises. Self-administered Thai massage has been reported to enhance local circulation, which may facilitate the removal of pain-inducing substances and increase tissue oxygenation, thereby supporting tissue recovery [25, 26, 46–48]. In addition, massage may stimulate cutaneous and deep mechanoreceptors, modulating nociceptive input through spinal and supraspinal inhibitory pathways, potentially contributing to reductions in pain sensitivity [46, 47, 49]. Stretching of the gastrocnemius and soleus together with plantar soft-tissue stretching may help restore intrinsic muscle flexibility, plantar fascia extensibility, and ankle dorsiflexion range of motion, which can lessen tensile load on the plantar fascia during gait and standing [15, 50–52]. Moreover, strengthening of the intrinsic foot muscles and plantar flexors may enhance medial longitudinal arch support and improve load distribution across the foot, thereby reducing mechanical stress on the plantar fascia [15, 53, 54]. These adaptations are consistent with the foot core paradigm, which highlights the stabilizing role of the small foot muscles, and with evidence supporting high-load resistance training in PF rehabilitation [15, 53, 54]. Taken together, these mechanisms provide a plausible physiological explanation for the improvements observed in the IA group. However, these mechanisms should be interpreted cautiously, as they were not directly measured in this study.

This study has certain limitations. First, the majority of the participants were older adults, which may restrict the generalizability of the findings to younger populations. Future research should therefore include participants across a broader age range to enhance external validity. Second, the follow-up period was limited to one month, which restricted the ability to evaluate the long-term effects of the intervention. Extended follow-up is recommended to determine the sustainability of treatment outcomes. Third, the participants were not blinded to the intervention they received. This lack of blinding is likely to have introduced performance and expectation bias, potentially leading to an overestimation of the treatment effects, especially for participant-reported outcomes. This limitation is largely inherent to the nature of physical and exercise-based interventions, for which participant blinding is often difficult to achieve. Nonetheless, future studies should consider strategies to implement participant blinding where feasible. Finally, the absence of a clinician-delivered comparison limits the ability to determine whether the improvements achieved through the self-administered program are equivalent to, or potentially less than, those produced by therapist-delivered treatments. Nevertheless, the favorable outcomes relative to self-care alone indicate that a structured self-administered approach can meaningfully reduce symptoms under minimal supervision. Future studies that directly compare self-administered and clinician-delivered interventions would help clarify their relative benefits and guide clinical decision-making.

## Conclusion

This study demonstrated that an IA program, incorporating self-administered Thai massage, stretching, and strengthening exercises, produced significantly greater improvements in pain intensity, pressure pain threshold, ankle dorsiflexion range of motion, and functional ability when added to basic self-care, compared with basic self-care alone in individuals with chronic PF. Importantly, no serious adverse events were reported, confirming the safety and feasibility of this supervised self-administered approach. By empowering patients to actively participate in their care while maintaining professional oversight, this model promotes safe and effective symptom management in community-based settings. Consequently, the IA program represents a promising, structured strategy that may reduce dependence on hospital-based treatment and enhance patient self-efficacy in the long-term management of chronic PF.

The favorable outcomes and safety profile also suggest that this supervised model may be adaptable into a fully independent, home-based format in future work, potentially improving accessibility and reducing the need for ongoing therapist involvement after the initial training phase.

## Supplementary Information

Below is the link to the electronic supplementary material.

Supplementary Material 1

## Data Availability

The dataset used in this study are available from the corresponding author on reasonable request.

## Abbreviations

PF: Plantar fasciitis
IA: Integrated self-administered program
FAAM: Foot and ankle ability measure
ANOVA: Analysis of variance
ANCOVA: Analysis of covariance
