# Supplementary material for: A randomized controlled trial of a supervised self-administered program for chronic plantar fasciitis
Source: Chiropr Man Therap. 2025 Dec 23;34:2. doi: 10.1186/s12998-025-00624-w (PMC12837117; doi:10.1186/s12998-025-00624-w)
Supplement: Supplementary file 1 — Supplementary Material 1 [file 12998_2025_624_MOESM1_ESM.docx]

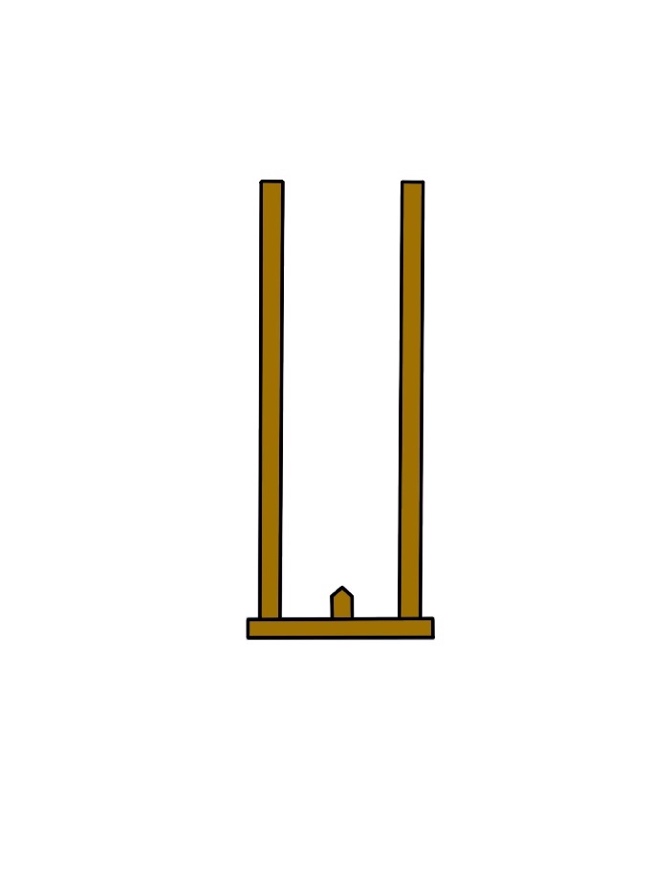


**Figure A1**. The self-treatment stick


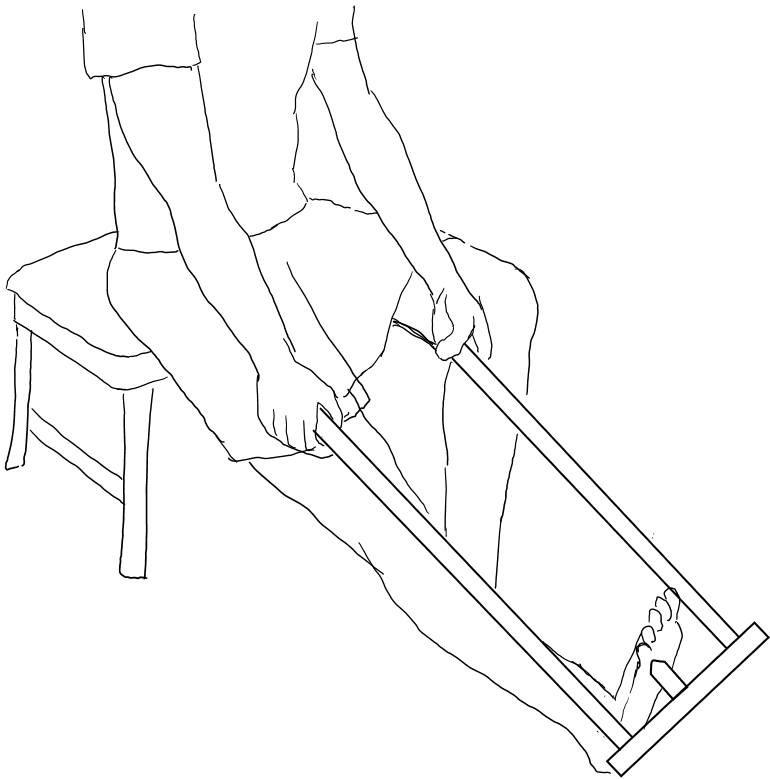


**Figure A2**. Self-administered foot massage performed with the self-treatment stick


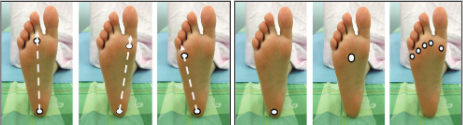


**Figure A3**. Specific lines and points for applying self-administered Thai massage on the plantar surface of the foot.


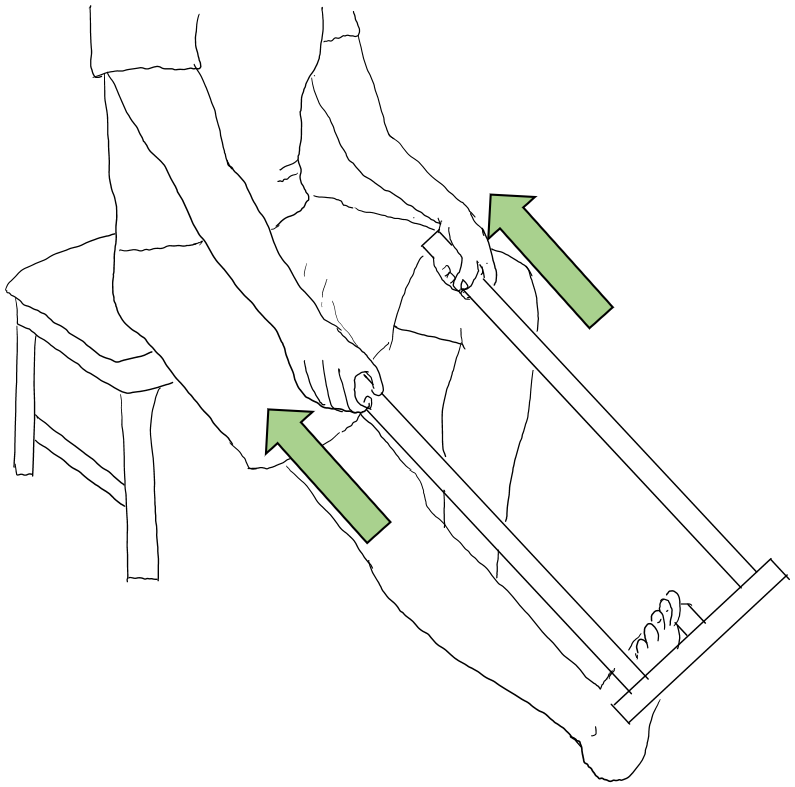


**Figure A4**. Self-administered gastrocnemius stretch performed with the self-treatment stick


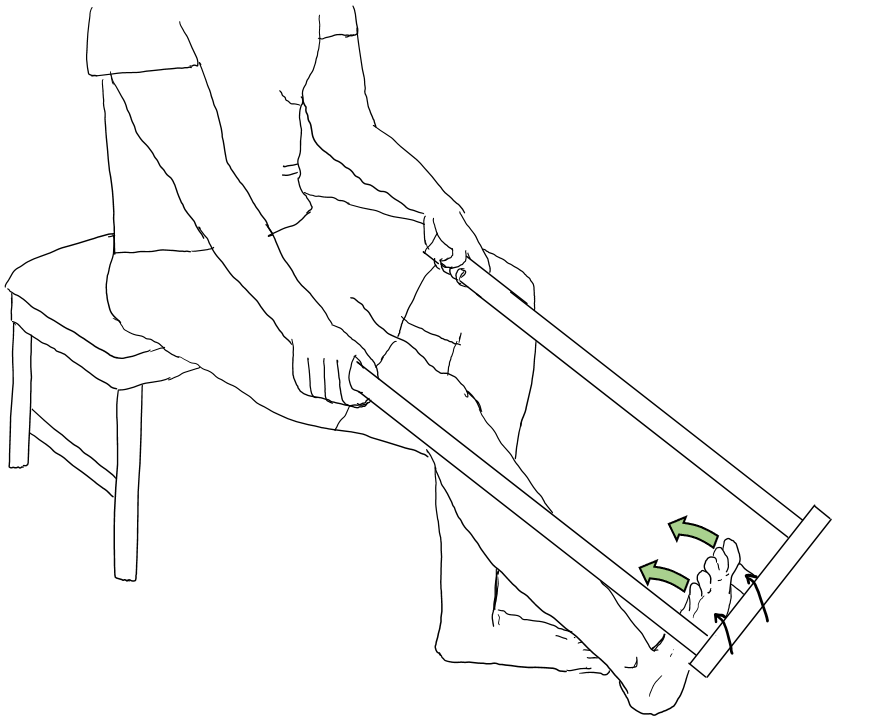


**Figure A5**. Self-administered soleus stretch performed with the self-treatment stick


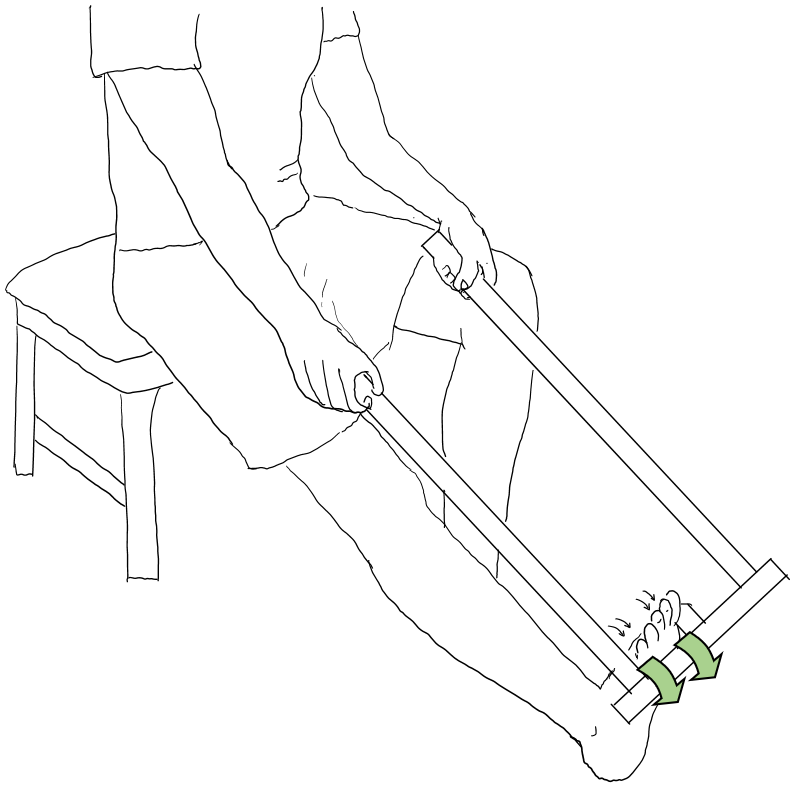


**Figure A6**. Self-administered exercise to strengthen the calf muscles performed with the self-treatment stick


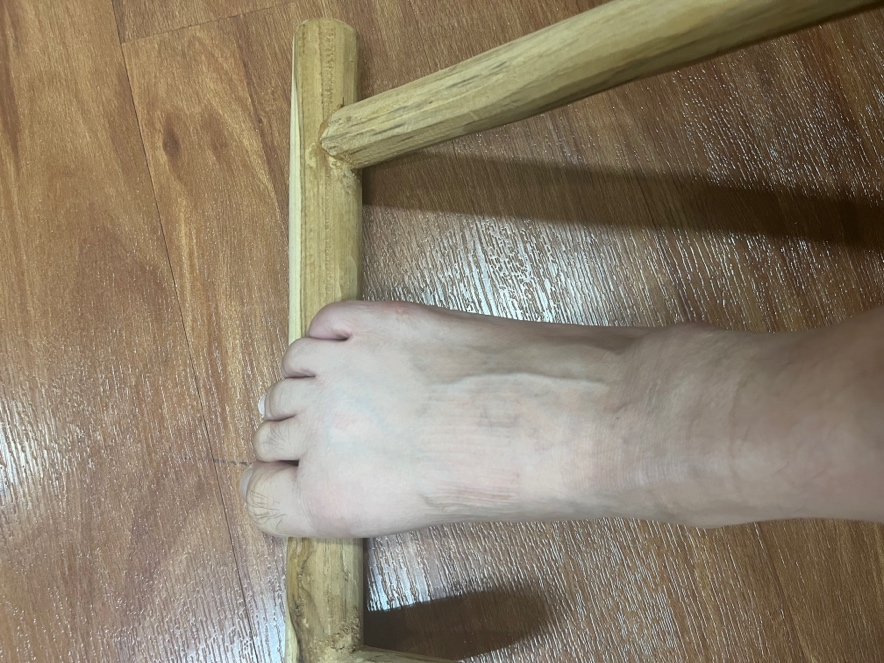


**Figure A7**. Self-administered toe strengthening exercise
